# The relationship between foveal outer nuclear layer thickness in the active and resolved phases of central serous chorioretinopathy treated with half-dose photodynamic therapy

**DOI:** 10.1186/s12886-019-1089-y

**Published:** 2019-03-29

**Authors:** Jia Yu, Yuan Lei, Qing Chang, Gezhi Xu, Xiaofeng Ye, Lei Li, Chunhui Jiang

**Affiliations:** 1 Department of Ophthalmology, Eye & Ear Nose Throat Hospital of Fudan University, 83 Fenyang Road, Shanghai, 200031 People’s Republic of China; 2 Shanghai Key Laboratory of Visual Impairment and Restoration, Fudan University, Shanghai, China; 3 NHC Key Laboratory of Myopia (Fudan University), Shanghai, China; 4 Laboratory of Myopia, Chinese Academy of Medical Sciences, Shanghai, China

**Keywords:** Central serous chorioretinopathy, Elasticity, Optical coherence tomography, Outer nuclear layer

## Abstract

**Background:**

To investigate the relationship between the foveal outer nuclear layer (ONL) thickness in the active and resolved phases of central serous chorioretinopathy (CSC), and its possible association with optical coherence tomography (OCT) parameters.

**Methods:**

The medical records of CSC patients treated with half-dose photodynamic therapy (PDT) between August 2011 and October 2017 were reviewed. The difference between the foveal ONL thickness at 12 m after half-dose PDT and that before half-dose PDT was analyzed, and its association with OCT parameters was assessed using generalized linear models.

**Results:**

Sixty-two patients were included. The mean difference in foveal ONL thickness was 9.15 ± 8.16 μm. The average ratios of the retinal detachment height to the subretinal space width on horizontal and vertical scans were 0.10 ± 0.04 and 0.12 ± 0.04, respectively. The ratio was independently associated with the degree of increase in the foveal ONL thickness difference on both the horizontal scans (β = 103.684, *P* = .000) and vertical scans (β = 67.569, *P* = .000), even after adjusting for potential confounders.

**Conclusions:**

The majority of resolved CSC eyes showed some increase in foveal ONL thickness, and the degree of increase was related to the ratio of the retinal detachment height to the subretinal space width in their active phase. It suggested that the retina is stretched when it becomes detached, and recovers with resolution of the subretinal fluid. Therefore, besides photoreceptor cell death, retinal stretch may contribute to the reduction in foveal ONL thickness in eyes with active CSC.

## Background

Central serous chorioretinopathy (CSC) is a common macular disease and often presents with well-circumscribed serous retinal detachment in the macular region on clinical examination, with one or several leakage points at the level of the retinal pigment epithelium (RPE), detectable with fluorescein angiography (FA) [1]. With the advent of optical coherence tomography (OCT), it is now possible to obtain high-resolution cross-sectional images of the retina in a noninvasive manner [2–4]. The typical pathological changes that occur in CSC, such as serous retinal detachment and RPE abnormalities, have been clearly demonstrated with OCT [5, 6]. It was recently reported that the thickness of the foveal outer nuclear layer (ONL) is significantly reduced in eyes with either active or resolved CSC [7–13],which has been speculated to attributed to photoreceptor cell death [7–9, 11, 14–16].

However, the relationship between the foveal ONL thickness in the active and resolved phases of CSC remains unclear. In this study, the difference between the foveal ONL thickness before half-dose photodynamic therapy (PDT) and 1 year after half-dose PDT is described, and its possible association with OCT parameters investigated.

## Methods

This study was approved by the Institutional Review Board of the Eye and ENT Hospital of Fudan University, and was performed in accordance with the principles of the Declaration of Helsinki. All of the subjects signed informed consent forms. The medical records of CSC patients treated with half-dose PDT in the clinic of the Eye & Ear Nose &Throat Hospital of Fudan University between August 2011 and October 2017 were reviewed.

### Patients

The clinical diagnosis of CSC was based on the following symptoms: reduced visual acuity, with or without metamorphopsia or micropsia; the presence of serous retinal detachment on both fundus and OCT examinations; the presence of active angiographic leakage on FA (TRC-50IX; Topcon Corp.,Tokyo, Japan); and/or abnormally dilated choroidal vasculature and other features on indocyanine green angiography (ICGA) (Spectralis HRA + OCT; Heidelberg Engineering, Heidelberg, Germany), consistent with the diagnosis of CSC [17, 18].

The data of the affected eyes were collected, including the symptom duration, best corrected visual acuity (BCVA), measured with a standard Snellen chart and converted to the logarithm of the minimum angle of resolution (logMAR) for statistical analysis, intraocular pressure, using a noncontact tonometer, central retinal thickness, half-dose PDT spot size, as well as the physical examination information of slit-lamp biomicroscopy.

The subjects included were those with clear and detailed medical records; symptom duration of 6 weeks or more; no amblyopia in the CSC eye; spherical equivalent between −2D and + 2D; no history of refractive surgery; subretinal fluid resolution within 2 months of half-dose PDT; no recurrence within 12 months of half-dose PDT; subretinal fluid involving the fovea; and a continuous external limiting membrane (ELM) at the fovea on all OCT images.

The exclusion criteria were: fragmentary medical records; symptom duration of less than 6 weeks; either clinical signs or a history of any other intraocular disease in the affected eye; any steroid use; inability to define the symptom duration before half-dose PDT; amblyopia in the CSC eye; spherical equivalent < −2D or > +2D; a history of refractive surgery; any residual subretinal fluid for up to 2 months after half-dose PDT; any recurrence within 12 months of half-dose PDT; any of staphyloma, choroidal excavation, or retinal dipping; RPE abnormality involving the fovea; subretinal fluid only at extra fovea; and an ELM interrupted at the fovea on any OCT image.

### Photodynamic therapy protocol

The half-dose PDT protocol for CSC was performed with half the normal dose of verteporfin (Visudyne; Novartis AG,Bülach, Switzerland), that is, 3 mg/m^2^verteporfin, based on the rationale that a lower dose has less-severe collateral damage effects to the retina and choroid. Verteporfin was infused over 8 min, followed by delivery of laser at 689 nm at 10 min from the commencement of infusion to target the area of choroidal dilation and hyperpermeability [19–21]. A total light energy of 50 J/cm^2^over 83 s was delivered to the angiographic leakage sites shown in FA or the area of choroidal hyperperfusion observed in ICGA [19–21].

### Optical coherence tomography protocol

All OCT images were obtained through a dilated pupil with a line scan protocol (line scans of 30°, composed of 100 averaged images**;** Heidelberg Spectralis OCT, Heidelberg Engineering, Heidelberg, Germany**)**. In each subject, this protocol was applied both vertically and horizontally and centered on the fovea in the CSC eyes. The OCT images (vertical and horizontal) that passed through the central fovea were selected for the measurement of the ONL thickness, as well as the height of retinal detachment, and the width of the subretinal space. OCT images taken within 1 week before half-dose PDT, 2 months (±2 weeks) after half-dose PDT, and 12 months (±2 weeks) after half-dose PDT were analyzed.

### Optical coherence tomography image analysis

**Fig. 1:**
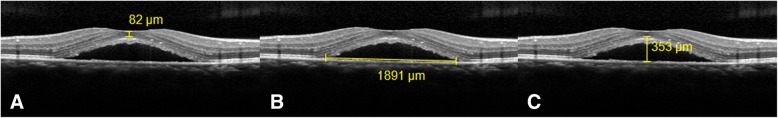
Illustration of optical coherence tomography (OCT) parameters in eyes with active central serous chorioretinopathy (CSC). **a**. Foveal outer nuclear layer(ONL) thickness was defined as the distance between the internal limiting membrane and the external limiting membrane (ELM) at the center of the fovea. **b.** Width of the subretinal space was defined as the distance between the vertices on the bottom side of the subretinal space on an OCT image. **c.** Height of the detached retina was defined as the vertical distance between the ELM and retinal pigment epithelium at the center of the fovea

The foveal ONL thickness was the average of the distances between the internal limiting membrane and the ELM at the center of the fovea measured from the horizontal and vertical scans respectively. (Figure 1a) The difference in the foveal ONL thickness was defined as the difference between the foveal ONL thickness measured at 12 m after half-dose PDT and that measured before half-dose PDT. The width of the subretinal space was defined as the distance between the vertices on the bottom side of the subretinal space on an OCT image(Figure 1b); and the height of the detached retina was defined as the average of the vertical distance between the ELM and RPE at the center of the fovea measured from the horizontal and vertical images respectively.(Fig. 1c) These measurements were made manually with the supplied caliper measurement tool (in 1:1 μm mode; HRA/Spectralis Viewing Module 6.0.9.0, Heidelberg Engineering). All the scans and measurements were made by Y.J. The repeatability of the measurements, including the foveal ONL thickness, the width of the subretinal space, and the height of the retinal detachment in the CSC eyes, were calculated from two horizontal scans taken of each eye during a single visit; 20 CSC eyes were included. Intraclass correlation coefficients (ICC) were used to assess the repeatability of the measurements (ICC values of 0.81–1.00 indicated almost perfect agreement between repeated measurements; values < 0.40 indicated poor to fair agreement) [22].

### Statistical analysis

The data were analyzed with SPSS for Windows version 21.0 (SPSS, Chicago, IL, USA). Descriptive statistics were calculated, including medians, means**,** proportions, and frequencies**.** The Kolmogorov–Smirnov test was used to confirm the normality of the data. Either Pearson’s correlation coefficient or Spearman’s correlation coefficient was used to examine the correlation between symptom duration and the foveal ONL thickness 12 months after half-dose PDT. A paired *t* test was used to examine the difference between the foveal ONL thickness 12 months after half-dose PDT and that before half-dose PDT. The associations of the foveal ONL thickness difference (dependent variable of interest) with the ratio of the retinal detachment height to the subretinal space width (independent variable) was assessed with generalized linear models. Factors such as sex, age, BCVA before and after half-dose PDT, and symptom duration before half-dose PDT were included in a multivariate model to adjust for potential confounding. A *P* value of < 0.05 was considered statistically significant.

## Results

Sixty-two patients were enrolled in the study. The demographic data for the patients and the values for their OCT parameters are listed in Table 1. Measurement of the foveal ONL thickness, the width of the subretinal space, and the height of the retinal detachment in the CSC eyes showed good repeatability, with ICC values of 0.977, 1.000, and 0.997. Symptom duration before half-dose PDT and foveal ONL thickness 12 months after half-dose PDT were negatively correlated (R = − 0.545, *P* = 0.000).

**Table 1:** The Demographic data for the patients and the values for their OCT parameters

	Mean	SD	Median	Min	Max	P*
Age(years)	**44.77**	9.08	43.00	31	69	0.649
Male, n (%)	49 (79.0%)	NA	NA	NA	NA	NA
Symptom duration before half-dose PDT (days)	132.05	156.17	**69.50**	42	784	0.000
BCVA before half-dose PDT (logMAR)	0.30	0.22	**0.20**	0.00	0.90	0.026
BCVA 12 m after half-dose PDT(logMAR)	0.06	0.14	**0.00**	0.00	0.70	0.000
CRT(μm)	**199.02**	110.04	189.50	31.00	468.00	0.910
Half-dose PDT spot size(mm^2^)	**7.57**	3.86	6.64	2.69	21.20	0.059
Height of retinal detachment(μm)	**284.23**	112.86	275.50	98.00	524.00	0.820
Width of subretinal space on horizontal scan (μm)	**2958.21**	1215.95	2742.50	962.00	7970.00	0.160
Width of subretinal space on vertical scan (μm)	**2665.98**	1296.03	2398.00	885.00	8566.00	0.068
H/W on horizontal scan	**0.10**	0.04	0.10	0.03	0.19	0.872
H/W on vertical scan	**0.12**	0.04	0.11	0.02	0.22	0.723
Difference of foveal ONL thickness (μm)	**9.15**	8.16	10.00	−8.00	29.00	0.922

*BCVA* best corrected visual acuity, *CRT* central retinal thickness, *N/A* not applicable; *H/W* the ratio of retinal detachment height to subretinal space width, *logMAR* logarithm of the minimum angle of resolution; *ONL* outer nuclear layer, *PDT* photodynamic therapy, *SD* standard deviation

*The Kolmogorov–Smirnov test was used to confirm the normality of the data. *P* ≥ 0.05 indicates that the data are normally distributed, and the mean value is shown in bold. *P* < 0.05 indicates that the data are not normally distributed, and the median value is shown in bold

**Fig. 2:**
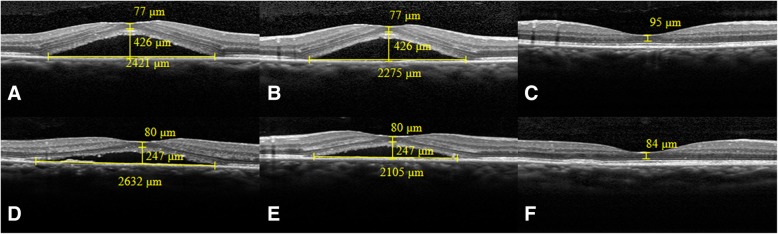
Foveal ONL thickness increased in resolved CSC eyes compared with thickness in their active phase (**a**-**f**). **a** and **b**. The horizontal and vertical line scans of a 48-year-old male patient with best corrected visual acuity (BCVA) of 20/25 and symptom duration of 42 days before half-dose photodynamic therapy (PDT). Ratio of the retinal detachment height to the subretinal space width was 0.176 on the horizontal scan and 0.187 on the vertical scan. **c.** The vertical line scan of the eye 12 months after half-dose PDT showing complete resolution of the subretinal fluid. The difference between the foveal ONL thickness12m after half-dose PDT and that before half-dose PDT was 18 μm. **d** and **e.** The horizontal and vertical line scans of a 55-year-old female patient with BCVA of 20/40 and symptom duration of 176 days before half-dose PDT. Ratio of the retinal detachment height to the subretinal space width on the horizontal scan was 0.094 and that on the vertical scan was 0.117. **f.** The horizontal line scan of the eye 12 months after half-dose PDT showing complete resolution of the subretinal fluid. The difference between the foveal ONL thickness 12 m after half-dose PDT and that before half-dose PDT was 4 μm

The majority of CSC eyes showed some increase in the foveal ONL thickness 12 months after half-dose PDT relative to that before half-dose PDT (Table 1:9.15 ± 8.16 μm; t = 8.83, *P* = 0.000; and Fig. 2). The Table 2 shows the linear regression analysis of the associations of OCT parameters (exposure variable of interest) against foveal ONL thickness difference (dependent variable) to calculate regression coefficients (β). In Model 1, without including the clinical characteristics, the ratio of the retinal detachment height to the subretinal space width was associated with the difference in foveal ONL thickness on both horizontal and vertical scans (Table 2: β = 130.484, *P* = 0.000,and β = 89.015, *P* = 0.000, respectively).

**Table 2:** The associations of optical coherence tomography parameters with difference of foveal outer nuclear layer thickness

	Model 1^a^		Model 2^b^	
	The difference of the foveal ONL thickness		The difference of the foveal ONL thickness	
	Beta(95% CI)	*P* Value	Beta(95% CI)	*P* Value
H/W in horizontal scan	130.484 (85.750, 175.217)	.000	103.684 (62.774, 144.593)	.000
H/W in vertical scan	89.015 (49.194, 128.837)	.000	67.569(31.864, 103.274)	.000

*CI* confidence interval, *ONL* outer nuclear layer, *H/W* the ratio of retinal detachment height and subretinal space width

^a^Unadjusted generalized linear model to determine the associations between the ratio (H/W) and the difference of foveal ONL thickness

^b^Generalized linear model adjusted for gender, age, best corrected visual acuity(BCVA) pre and after half-dose photodynamic treatment (PDT), and symptom duration before half-dose PDT to determine the associations between the ratio (H/W) and the difference of foveal ONL thickness

In Model 2, to control for the potential confounding effects of clinical characteristics, we included sex, age, symptom duration before half-dose PDT, and BCVA before and after half-dose PDT. The results remained similar after adjustment for these clinical characteristics (Table 2: β = 103.684, *P* = 0.000 for horizontal scans; and β = 67.569, *P* = 0.000 for vertical scans).

## Discussion

The study showed that the foveal ONL thickness increased in the majority of resolved CSC eyes, and the degree of increase was associated with the ratio of the height of the retinal detachment to the width of the subretinal space.

Previously, Ohkuma has observed that the foveal ONL thickness increased by about 11 μm in some resolved CSC eyes compared with that in their active phase, which is consistent with our findings [12].Although the exact mechanism of the increase in ONL thickness remains unclear, the following scenario is possible. Normally, the retina is attached to the RPE, thus, the retina and the underlying RPE are the same in length. While, in CSC, the hyperpermeability of the choroid causes hydrostatic pressure increase and leakage through the RPE rupture, resulting in neurosensory retinal detachment [23].On an OCT image, the border of the subretinal space appears approximately as a triangle: the underlying RPE forms the base of the triangle, i.e., the bottom side, the outer border of the detached retina forms the other two sides, and the fovea is the top vertex of the triangle. Based on triangle inequality, the outer border of the detached retina is longer than the underlying RPE. Thus, we speculate that the retina is stretched when it is detached, and then recovers when it reattaches to the RPE. Previously, the elastic properties of the human retina have been examined in donated eyes under physiological conditions [24].Moreover, in this study, the association between the ratio of retinal detachment height to subretinal space width and the increase degree of foveal ONL thickness difference further support the hypothesis.

Furthermore, based on the above hypothesis, the morphological character of the subretinal space that the fovea always appears to be the top vertex of the triangle can be explained as follows. The fovea, which consists mainly of photoreceptors, is the thinnest part of the retina with only one horizontal layer of ELM. In contrast, the parafoveal region consists of multiple layers, including the outer plexiform layer, inner plexiform layer, and retinal nerve fibre layer. This retinal architecture may make the foveal region mechanically weak; therefore, it will stretch more.

Previously, it has been speculated that the reduction in ONL thickness after retinal detachment is attributed to photoreceptor cell death through apoptosis, necroptosis, autophage, and macrophage or microglial infiltration [7–9, 11, 14–16].The present study showed that besides the photoreceptor cell death, retinal stretch may contribute to the foveal ONL thickness reduction in eyes with active CSC. This suggests that photoreceptor cell death is overestimated, especially in eyes with high ratio of retinal detachment height to subretinal space width. This study had several limitations: 1) the inference of retinal stretch within the detached area still requires firm evidence; 2) the lack of a normal control group, a standard dose control group, and the measurement of ONL by a second blinded examiner; and 3) the sample group was small. Basic research with a proper animal model may provide further firm evidence. Further prospective studies with large populations and control groups may tell us more.

## Conclusions

The majority of eyes with resolved CSC showed some increase in the foveal ONL thickness, and the degree of increase was related to the ratio of the retinal detachment height to the subretinal space width in the active phase. When estimating the photoreceptor cell death by the reduction of foveal ONL thickness in eyes with active CSC, the retinal stretch should be taken into consideration.

## Abbreviations

BCVA: Best corrected visual acuity
CSC: Central serous chorioretinopathy
ELM: External limiting membrane
FA: Fluorescein angiography
ICCs: Intraclass correlation coefficients
ICGA: Indocyanine green angiography
logMAR: Logarithm of the minimum angle of resolution
OCT: Optical coherence tomography
ONL: Outer nuclear layer
PDT: Photodynamic therapy
RPE: Retinal pigment epithelium
